# Progress of tanshinone IIA against respiratory diseases: therapeutic targets and potential mechanisms

**DOI:** 10.3389/fphar.2025.1505672

**Published:** 2025-02-24

**Authors:** Zhaohui Ding, Youlin Deng, Huie Luo, Cuiwang Liu, Minjuan Yang, Hanrong Xue, Zhengtao Chen

**Affiliations:** ^1^ Department of Clinical Medicine, Jiangxi University of Traditional Chinese Medicine, Nanchang, China; ^2^ Affiliated Hospital of Jiangxi University of Traditional Chinese Medicine, Nanchang, China

**Keywords:** tanshinone IIA, phytomedicine, respiratory disease, pharmacological mechanisms, molecular targets

## Abstract

The respiratory system stands as one of the eight pivotal systems within the human body, responsible for a range of essential functions. Primarily, it facilitates the absorption of oxygen from the external environment and the expulsion of carbon dioxide, thereby playing a crucial role in regulating the body’s acid-base balance. Furthermore, it helps to maintain the stability of the internal environment, ensuring the smooth progression of normal metabolic processes and sustaining life activities. In the wake of the novel coronavirus pneumonia outbreak, respiratory diseases have continued to exhibit comparatively high morbidity and mortality rates, underscoring the urgent need for the discovery of novel therapeutic agents. Tanshinone IIA (Tan IIA), a bioactive chemical constituent derived from *Salvia miltiorrhiza Bunge,* has emerged as a promising candidate. As a significant fat-soluble compound, Tan IIA has traditionally been utilized in the treatment of cardiovascular diseases. As research on Tan IIA has progressed, its multifaceted therapeutic potential has been unveiled. Specifically, Tan IIA has demonstrated anti-inflammatory, anti-oxidative stress, anti-fibrosis, and anti-cancer effects. In recent years, a wealth of studies has concentrated on elucidating its impact on various respiratory diseases, including asthma, chronic obstructive pulmonary disease, pulmonary hypertension, pulmonary fibrosis, acute lung injury/acute respiratory distress syndrome, and lung cancer. These findings collectively suggest that Tan IIA holds considerable promise in the realm of anti-respiratory disease therapies. The present article undertakes a comprehensive review of the targets and potential mechanisms of Tan IIA against respiratory diseases, offering valuable insights that can serve as a reference for future research endeavors and clinical applications of Tan IIA in the treatment of respiratory ailments.

## 1 Introduction

The respiratory system, including the nose, pharynx, larynx, trachea, bronchi, and lungs, is the gateway for communication between the body and the outside world (Frohlich, 2024). Respiratory diseases mainly include pneumonia, chronic obstructive pulmonary disease (COPD), pulmonary hypertension (PH), pulmonary fibrosis (PF), acute lung injury/acute respiratory distress syndrome (ALI/ARDS) and lung cancer. According to statistics, chronic respiratory diseases were the third leading cause of death among four million people in 2019, with approximately 262.4 million prevalent cases of asthma and 37 million new cases in 2019, while there were approximately 223 million prevalent cases of COPD and 16.2 million new cases in 2019. An additional study showed that the overall prevalence of interstitial lung disease was proximately 6.3–76.0 cases per 100,000 people (Wijsenbeek et al., 2022). Lung cancer is the most common cause of cancer death, accounting for approximately 18.4% of total cancer deaths (Bray et al., 2018). Because of the high prevalence and poor prognosis of respiratory diseases, the treatment and prevention of respiratory diseases are increasingly important. At present, most of the drugs for the treatment of respiratory diseases are anti-infective, anti-tussive, anti-asthmatic, expectorant, and anti-inflammatory (Ambrosino and Fracchia, 2019; Molassiotis et al., 2010). In recent years, with the important therapeutic role of traditional Chinese medicine being demonstrated in the epidemic situation of novel coronavirus pneumonia (Lyu et al., 2021; Zhang et al., 2021), there have been more and more preclinical studies of traditional Chinese medicine against respiratory diseases.

**FIGURE 1:**
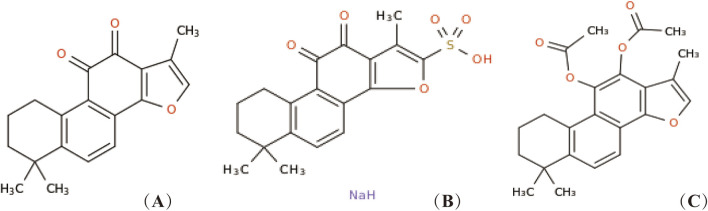
Chemical structure of Tan IIA and its derivatives. Tan IIA **(A)**, STS **(B)**, generates acetyl Tan IIA **(C)**.

*Salvia miltiorrhiza Bunge*, the dried root or rhizome of perennial erect herbs in the Labiatae family, has the effects of promoting blood circulation and removing blood stasis to relieve pain, clearing the heart to remove annoyance and tranquilize the mind, and cooling blood to eliminate carbuncle (Meim et al., 2019). Tan IIA, a major liposoluble component of *S. miltiorrhiza Bunge* belonging to diterpenoid quinones (Jiang et al., 2019), has been widely used as a natural medicine for the treatment of various diseases, including cardiovascular diseases (Ren et al., 2019), renal diseases (Chen et al., 2023), neurodegenerative diseases (Subedi and Gaire, 2021), and tumor-related conditions (Fang et al., 2020). In addition to *S. miltiorrhiza*, the roots of other plants such as *Salvia sclarea* L. and *Salvia przewalskii* Maxim also contain Tan IIA. Figure 1 depicts the chemical structure of Tan IIA. It exhibits diverse pharmacological activities, including anti-inflammatory, antioxidant, anti-fibrotic, anti-apoptotic, anti-tumor, anti-myocardial injury, antibacterial, analgesic, vasodilatory, endothelial protection, and immunomodulatory (Tang and Zhao, 2024; Huang B. et al., 2022; Ansari et al., 2021). However, Tan IIA has limitations such as poor water solubility, an extensive first-pass effect, and low bioavailability (Wang R. et al., 2019; Yan et al., 2015).To enhance its biological activity, structural modifications have been made, primarily at position 16 of the furan ring (Huang X. et al., 2022). For instance, sodium tanshinone IIA sulfonate (STS) is generated through sulfonation at this position, resulting in improved water solubility and bioavailability compared to Tan IIA (Zhou et al., 2019; Tian and Wu, 2013). STS has been used as an alternative to Tan IIA in numerous studies and has shown efficacy in treating coronary heart disease, angina pectoris, and myocardial infarction (Wu et al., 2023; Tan et al., 2018). Previous studies have also explored C-loop modifications of Tan IIA, such as the reduction of ortho-quinone at positions 11 and 12 followed by acetylation, resulting in acetyl Tan IIA (ATA) with increased water solubility and anti-tumor activity (Tian et al., 2010). While many studies have investigated the potential of Tan IIA against respiratory diseases, a systematic summary of its effects in this context is still lacking.

In this paper, we summarize the preclinical studies of Tan IIA in the treatment of asthma, chronic obstructive pulmonary disease, pulmonary hypertension, pulmonary fibrosis, acute lung injury/acute respiratory distress syndrome, lung cancer, and other respiratory diseases (Table 1). We mainly focus on summarizing the targets and potential mechanisms of its therapeutic effect, providing a theoretical basis for further pharmacological study and clinical application of Tan IIA in the treatment of respiratory diseases, and offering new insights into its future development direction.

**TABLE 1:** The role of Tan IIA in various respiratory diseases.

Disease	Models and modeling methods	Research type	Tan IIA or derivatives dose and treatment Schedule	Targets	References
asthma	mouse tracheal ring; CCh (1 µM) stimulates for 15–20min	*In vitro*	STS:10,50,100 µM for 10 min	↓LCC, ↑KATP, ↓Ca^2+^ in ASMCs	Zhang et al. (2017)
	BALB/c mice [female, n = 70]; OVA (50 μL) combined with Al(OH)_3_ (50 μL) i.p. for d0 and d14, then inh 1%OVA 30min/qd from d21 to d23	*In vivo*	Tan IIA:10 mg/kg/d i.p. bid from d18 to d23	↓NF-κB, ↑HO-1, ↑Nrf, ↑GPx, ↑SOD, ↓MDA in lung tissue; ↓IL-4, ↓IL-5, ↓IL-13 in BALF	Wang et al. (2019a)
	BALB/c mice [female, n = 40]; OVA (50 μL) combined with Al(OH)3 (1 mg) i.p. for d0 and d14, then inh 1%OVA 30min/qd from d28 to d30	*In vivo*	Tan IIA:5,10 mg/kg/d i.p. d28∼d30 before inh 30min	↓Mucin in bronchia; ↓IL-4, ↓IL-5, ↓IL-13, ↓INF-γ mRNA in BALF	Heo and Im (2019)
	Rat RBL-2H3 mast cells; incubated with Anti-DNP IgE for 18h	*In vitro*	Tan IIA:1,3,5,10 µM for 30min in PIPES buffer at 37 °C	↓β-amino-hexosidase, ↓mast cell degranulation in cell	
COPD	Kunming mouse tracheal epithelium; amiloride (100 μM) stimulates for 40s; mouse tracheal epithelial cells	*In vitro*	STS:0.1,0.3,1,3,6,10,30,60,100,300 μM for 40s	↑CaCC, ↑mAChR, ↑Ca2+, ↑Cl- in cell; ↑Isc in tracheal epithelium	Chen et al. (2017)
	C57BL/6 J mice [male, n = 12]; Inhaling LPS(7.5μg/mice) through the nose for d1 and d14, and total body exposure to CS was nine cigarettes per hour for 2 hours, bid, 6 days a week, for 60 days	*In vivo*	STS:5 mg/kg/bid inhale for 30min before daily exposure to CS for 60 days	↑CFTR, ↓ERK1/2, ↓NF-κB p65, ↓IL-6, ↓IL-8 in the lung tissue and 16HBE	Li et al. (2018)
	16HBE; 2%CSE induced for48h	*In vitro*	STS:10 μg/ml pre-treatment 2h		
	C57BL/6J mice [male, n = 24]; LPS(7.5 µg) are injected into the trachea for d1 and d14, and total body exposure to CS was nine cigarettes per hour for 4 hours, bid, 6 days a week, for 3 months	*In vivo*	STS:5 mg/kg/bid inh before daily exposure to CS	↓MAPK, ↓HIF-1α, ↓TNF-α, ↓IL-1β, ↓ROS, ↓HO-1, ↓NOX1 in the lung tissue and RAW 264.7 cells; ↓p-ERK, ↓p38 MAPK, ↓JNK in RAW 264.7 cells	Guan et al. (2018)
	mouse macrophage RAW 264.7 cells; CSE (2%)-induced for 24h	*In vitro*	STS:1,2.5,5 µM incubate for 24h		
	C57BL/6J mice [male, n = 50]; CS-induced for 90days, then plus LPS(1ug/per) nasal drops on d91	*In vivo*	STS:10 mg/kg/d i.p for 5 consecutive days (d92-d96)	↓NF-κB, ↓p-ERK1/2, ↓NF-κB p65, ↓IL-6, ↓IL-8 in the lung tissue and 16HBE	Li et al. (2020)
	16HBE cell; CSE (2%)-induce for 48h, then LPS (10 μg/ml) treatment	*In vitro*	STS:10 μg/ml 2h prior to 24 h or 30 min of LPS treatment		
	alveolar epithelial A549 cells; CSE (3%) induced for 48h	*In vitro*	STS:1,2.5,5uM incubate for 48 h	↑SIRT1, ↑DWM, ↑Mitochondrial respiratory chain complex IIV, ↑Pink1, ↓ROS, ↑Mfn2, ↓Drp1, ↓caspase-3 in A549 cells	Guan et al. (2021)
PH	Adult SD rats [male, n = 48]; placed intermittently in a low-pressure anoxic chamber (380 mmHg,10%O_2_, 8h) daily and then exposed to 21% oxygen for 2–3 weeks	*In vivo*	STS:10 mg/kg/d i.p. for 21d	↑Kv2.1 in PASMC	Huang et al. (2009)
	the healthy rat’s PASMC; Hypoxia-induced (93% N_2_, 5% CO_2_ and 2% O_2_)	*In vitro*	STS:0,2,10,25,50 μg/mL for 24 h		
	Adult SD rats [male, n = 48]; placed intermittently in a low-pressure anoxic chamber (380 mmHg,10%O2, 8h) daily and then exposed to 21% oxygen for 4 weeks	*In vivo*	Tan IIA:10 mg/kg/d, i.p. after hypoxic exposure lasted for 4 weeks	↑Ikv, ↓VDC, ↓Cyt,↑Kv1.5,↑Kv2.1,↑Oxygen sensitive voltage-gated potassium channel in PASMC	Zheng et al. (2015)
	Adult SD rats [male, n = 48]; remained in a low-pressure anoxic chamber (380 mmHg, 10%O2) for 4 weeks		Tan IIA:10 mg/kg/d, i.p. 2weeks		
	PASMCs; Hypoxia induction	*In vitro*	Tan IIA:5,25 μM/ml		
	Adult SD rats [male, n = 28]; Put in a hypoxic chamber (10%O_2_) for 21day, then use MCT (50 mg/kg) i.h once	*In vivo*	STS:10 mg/kg/d i.p. for 21d	↓TRPC1, ↓TRPC6, ↓SOCE in PASMCs anddistal pulmonary artery and pulmonary smooth muscle	Wang et al. (2013a)
	Rat Distal PASMCs; Hypoxic (4% O_2_) exposure for 60h	*In vitro*	STS:0,0.1,1,12.5,25 µM incubate for 60h		
	Adult SD rats [male, n = 40]; Placed in hypoxic chamber (10% ± 1% O_2_) for 21 days	*In vivo*	STS: 30 mg/kg/d i.p. for 21d	↑PKG, ↑PPAR-γ, ↓TRPC, ↓SOCE in PASMC and distal pulmonary artery	Jiang et al. (2016)
	rat PASMCs; hypoxia (4%O_2_) induced for 60h	*In vitro*	STS:0, 12.5 µM incubate for 35min		
	the male Sprague-Dawley rat’s PASMCs; Hypoxia-induced (3% O2)	*In vitro*	Tan IIA:0,3,10,30,50 μg/mL for 24h	↓Skp2, ↓p-Akt, ↑P27 in PASMC	Luo et al. (2013)
	Adult SD rats [male, n = 32]; placed in a low-pressure anoxic chamber (380 mmHg,10%O2) for 4 weeks	*In vivo*	Tan IIA:10 mg/kg/d, i.p. for 28d		
	Adult SD rats [male, n = 50]; placed in a hypoxic chamber (10%O_2_, 8 h/d) and then returned to normal atmospheric conditions (16 h/d) for 3 weeks	*In vivo*	STS:10,30 mg/kg/d, i.p. after hypoxic lasted for 3 weeks	↓PI3K, ↓p-Akt, ↓mTOR, ↓p-mTOR, ↓S6K1, ↓p-S6K1, ↓IL-6, ↓IL-8, ↓TNF-α in the lung tissue	Bao et al. (2020)
	Adult SD rats [male, n = 24]; placed on normal hypoxia (10%O_2_) for 21d	*In vivo*	STS:30 mg/kg/d, i.p. for 21d	↓α-SMA, ↓VWF, ↓Bax↓Caspase3 in Pulmonary vascular endothelial cells; ↑BMPR2, ↑p-Smad1/5/9 in the lung tissue	Wang et al. (2022)
	Rat PMVECs; placed on normal hypoxia (4%O_2_) for 72h	*In vitro*	STS:12.5 µM incubate for 72h	↑BMPR2, ↑p-Smad1/5/9 in cell	
	HESC-ECs; placed on normal hypoxia (4%O_2_) for 48h		STS:12.5 µM incubate for 48h		
PF	Adult SD rats [male, n = 30]; BLM (50 μL,5 mg/kg) endotracheal in a single drug delivery	*In vivo*	Tan IIA:25 mg/kg/d, i.p. for 28d	↑ACE2, ↑Ang-(1–7), ↓TGF-β1 in the lung tissue	Wu et al. (2014)
	Adult SD rats [male, n = 100]; BLM(5 mg/kg) endotracheal in a single drug delivery	*In vivo*	Tan IIA:15 mg/kg/d, i.p. for 28d	↓TNF-β, ↓IL-1β, ↓IL-6 in BALF; ↓iNOS, ↓No, ↓COX-2, ↓PGE2, ↓MDA in the lung tissue	He et al. (2015)
	Adult SD rats [male, n = 36]; BLM(5 mg/kg) endotracheal in a single drug delivery	*In vivo*	Tan IIA:15 mg/kg/d, i.p. for 28d	↓TGF-β, ↓p-Smad-2/3, ↓Type I Collagenase, ↓α-SMA, ↓FN, ↓Vimentin, ↓EMT in the lung tissue and A549 cells; ↓CD-68 in the lung tissue	Tang et al. (2015)
	human alveolar epithelial A549 cells; TGF-β1 (10 ng/mL) induced for 48h	*In vitro*	STS:10 µM incubate for 2h		
	Adult SD rats [male, n = 48]; silica dust suspension (1 mL 50 mg/mL) endotracheal in a single drug delivery	*In vivo*	Tan IIA:25 mg/kg/d, i.p. for 40d	↓TNF-α, ↓IL-6, ↓IL-1β,Type I collagen, ↓FN,↓α-SMA, ↓TGF-β1,↓p-Smad2/3, ↓p-Smad3, ↑Smad7, ↓NOX4, ↑NRF2, ↑HO-1, ↑NQO-1 mRNA, ↓ROS,↓MDA,↑SOD, ↑GSH-Px in the lung tissue	Feng et al. (2019)
	Adult SD rats [male, n = 36]; silica dust suspension (1 mL 50 mg/mL) endotracheal in a single drug delivery	*In vivo*	Tan IIA:25 mg/kg/d, i.p. for 40d	↓Type I collagen, ↓FN,↓α-SMA, ↓TGF-β1 in the lung tissue	Feng et al. (2020)
	Human lung adenocarcinoma A549 and human bronchial epithelial cells (HBE); SiO2 (100 μg/m L) induced for 24h	*In vitro*	Tan IIA: 5,10,20 μM incubate for 24h	↓TGF-β1, ↓p-Smad3, ↑Smad7, ↑Nrf2, ↑HO-1, ↑NQO-1, ↓EMT in A549 and HBE cells	
	The human acute monocytic leukemia cell line THP-1; LPS(0.5 μg/ml) induced for 24h	*In vitro*	STS:0,5,10,20,50,100,200,300,400,500,600 μg/ml, pretreatment of 2 h, join again after LPS treatment for 24 h	↓IL-1β, ↓TNF-α, ↓FMT in THP-1 cells	Jiang et al. (2020)
	fibroblast cell line MRC-5 cell; TGF-β1 (10 ng/ml) induced for 48h		STS:0,50,60,75,100,150,200,225,250 μg/ml, pretreatment of 4 h, join again after LPS treatment for 24 h	↓COL1α1, ↓α-SMA, ↓FMT in MRC-5 cell	
	Wistar rats [male, n = 72]; silica dust suspension (1 mL 50 mg/mL) endotracheal in a single drug delivery	*In vivo*	STS: 25 mg/kg/d, i.p for 28d	↓Type I collagen, ↓Type III collagen, ↓hydroxyproline, ↓ROS, ↓MDA, ↑Nrf2, ↑Trx,↑TrxR in the lung tissue	Zhu et al. (2020)
	Adult SD rats [male, n = 18]; BLM (2.5 U/kg) endotracheal in a single drug delivery	*In vivo*	Tan IIA:25 mg/kg/d, i.p, for 21d	↓Zbtb16, ↓Type I collagen, ↓fibronectin, ↓α-SMA, ↓p-Smad2/3 in the lung tissue and MRC-5 cell; ↓HYP in the lung tissue	Zhang et al. (2024a)
	fibroblast cell line MRC-5 cell; TGF-β1 (10 ng/ml) induced for 24h	*In vitro*	Tan IIA:10 μM incubate for 24h		
ALI/ARDS	KM mice [female, n = 128]; LPS (15 mg/kg), i.p. once	*In vivo*	Tan IIA:10,30,50 mg/kg, i.p. and before LPS attack 0.5h	↓NF-KB, ↓PLA2 in cell; ↓MPO in the lung tissue	Xu et al. (2009)
	The alveolar macrophage cell line NR8383 and human lung adenocarcinoma epithelial cell line A549	*In vitro*	Tan IIA:0,5,10,20 μg/mL incubate for 24h		
	BALB/c mice [male, n = 32]; LPS (10 mg/kg), i.p. once	*In vivo*	Tan IIA:10 mg/kg, i.p. and before LPS attack 0.5h	↓PI3K, ↓AKT, ↓MAPK, ↓HIF-1a in cell; ↓TNF-α, ↓IL-1β, ↓IL-6 in the lung tissue	Xu et al. (2011)
	macrophage cell lines (NR8383 and RAW 264.7)	*In vitro*	Tan IIA:1,5,10,20 μg/mL incubate for 30 min		
	Adult SD rats [male, n = 30]; PQ (35 mg/kg) endotracheal in a single drug delivery	*In vivo*	Tan IIA:25 mg/kg/d, i.p. for 3d	↑ACE2, ↑Ang-(1–7) in the lung tissue; ↓LDH, ↓MPO in BALF	Wang et al. (2018)
	C57BL/6J mice [/, n = 20]; LPS (15 mg/kg), i.p. once	*In vivo*	Tan IIA:10 mg/kg/d, i.p. for 3d and before LPS attack 0.5h	↑Sirt1, ↓NF-kB p65, ↓TNF-α, ↓IL-1β, ↓IL-6, ↓MPO in the lung tissue	Quan et al. (2019)
	BALB/c mice [female, n = 30]; 50 μL LPS (2 mg/kg) i.p. once	*In vivo*	Tan IIA:10 mg/kg/d, i.p. for 3d	↓IL-6, ↓IL-8, ↓TNF-α in the lung tissue	Zhao et al. (2022)
	MAECs, MMAs; LPS (10 ng/m L) induce 24h	*In vitro*	Tan IIA:100 ng/mL, incubate for 24h	↑M2, ↓M1, ↓NF-kB p65, ↓HIF1α in cell	
	Adult SD rats [male, n = 15]; Cecal ligation and puncture (CLP) to establish sepsis in rats	*In vivo*	Tan IIA: 5,10,20 mg/kg, i.p. after CLP modeling 6 h	↓ROCK2, ↓Bax, ↓cleaved caspase-3, ↓IL-1β, ↓TNF-α, ↓IL-6 in the lung tissue	Liu et al. (2022)
	RLE-6TN cells; LPS (100,200,400 ng/mL) induce 24h	*In vitro*	Tan IIA:10 μM pretreatment for 30min		
	C57BL/6 mice [male, n = 50]; blast (321 ± 24PSI) shock to the chest induced	*In vivo*	Tan IIA:30 μg/kg, i.p. before impact injury 1h	↓p-PI3K, ↓p-Akt, ↑p-FoxO1, ↑Bcl2, ↓Bax, ↓Caspase-3, ↓ROS, ↓MDA5↓IRE-α, ↑SOD-1, ↓TNF-α, ↓IL-6, ↓IL-1β, ↑IL-10 in the lung tissue	Liu et al. (2020)
	Adult SD rats [male, n = 120]; High-fat diet for 8 weeks; the right kidney was resected, the left renal artery was clamped for 30min, and the renal artery was reperfused for 24h	*In vivo*	Tan IIA:5,10 mg/kg/d, i.p, before renal ischemia-reperfusion for 2 weeks	↑PI3K, ↑p-Akt, ↑p-Bad,↑Bcl-2, ↓Bax, ↓Cyt-c, ↓caspase-3, ↓PARP in the lung tissue; ↑DRp1, ↓Mfn1, ↓Mfn2, ↑PGC-1α, ↑NRF1, ↑TFAM, ↓ROS, ↓MPTP, ↑RCR, ↑ATP in mitochondria of lung tissue	Tai et al. (2021)
	C57BL/6 mice [male, n = 25]; ischemia-reperfusion 48 h	*In vivo*	Tan IIA:30 μg/kg, i.p. before injury 1 h	↑p-PI3K, ↑p-Akt, ↑p-mTOR, ↑Bcl2, ↓Bax, ↓Bim, ↓Bad, ↓Caspase3, ↑Gpx4, ↑SLC7A11, ↑GSH, ↓Ptgs2, ↓MDA, ↓TNF-α, ↓IL-6, ↓IL-1β, ↑IL-10, ↓MPO, ↑SOD in the lung tissue	Zhang et al. (2023)
lung cancer	The human non-small cell lung cancer A549 cell line	*In vitro*	Tan IIA:80,60,40,30,20,15,10,5,2.5 μmol/L, incubated for 24, 48 and 72h, respectively; Tan IIA (31 μmol/L) and adriamycin (2.5 μmol/L) were treated together for 24 h	↓VEGF, ↓VEGFR2 in cell	Xie et al. (2015)
	Human NSCLC cells, including HCC827, H1975, and A549	*In vitro*	Tan IIA:0,1,2,5 μM culture for 24,48 and 72h respectively	↓p-EGFR, ↓Akt, ↓EKR1/2, ↑ubiquitination of Mcl-1 in cells and the lung tissue	Gao et al. (2020)
	athymic nude mice [female, n = 12]; HCC827 cells were inoculated subcutaneously into the right abdomen of mice	*In vivo*	Tan IIA:10 mg/kg/d i.p. for 30d, when the tumor volume reached about 100 mm^3^		
	A549、NCI-H1975	*In vitro*	Tan IIA:0,30,40 µM (A649 was treated with 30 µM and H19 with 40 µM) culture for 24h	↓CCNA2, ↓CDK2, ↓AURKA, ↓PLK1, ↓ERK, ↓CCNA2-CDK2 complex in cells	Li et al. (2021)
	Human NSCLC cell lines A549, H460, H1299, and human NK cells	*In vitro*	Tan IIA:0.078,0.156,0.3125,0.625,1.25,2.5,5,10,20 μM incubated for 24,48,72 h	↑p-PERK, ↑ATF4, ↑CHOP, ↑ULBP1, ↑DR5 in A549, H460 and H1299; ↑CD107α in NK cells	Sun et al. (2021)
			Tan IIA: 0,2.5,10 μM incubated for 24 h, then cultivating 4 hours together with the NK cells		
	SCID-Bg mice [male, n = 20] with H460 cell subcutaneous xenograft tumors; NK cells were injected at day 1, 8 and 15 respectively	*In vivo*	Tan IIA:0.5 mg/kg, i.p. every 2 days starting on day 3		
	C57BL/6 mice [male, n = 20] were inoculated with LLC cells subcutaneously on the right back; PK136 antibody inject-ted on days 0, 3, 7, 10 and 17 respectively		Tan IIA:0.5 mg/kg, i.p. every 2 days starting on day 3		
	Human NSCLC A549 and H292 cell lines	*In vitro*	Tan IIA:0.25,0.5,1,2,4,8,16,32 µM incubated for 48h	↓SIX1, ↓PKM2, ↓HK2,↓LDHA in cells and the lung tissue	Qi et al. (2022)
	BALB/c-nu/nu nude mice [male, n = 12]; A549 cells were injected subcutaneously into nude mice	*In vivo*	Tan IIA: 20 mg/kg/d, i.p. for 2weeks		
	Human NSCLC A549 and H292 cell lines	*In vitro*	Tan IIA:0,5,10,20,40 μM incubated for 48h	↓CIRC 0020123, ↓HMGB3, ↑miR-1299 in cells	Sun et al. (2023)
	LUAD cell lines (A549 and H1299)	*In vitro*	STS:0,10,50,100,200 μM incubated for 24h	↑miR-874, ↓eEF-2K, ↓TG2 in cells	Wang et al. (2023)
	Balb/c-nu/nu nude mice [male, n = 36]; A549 cells were injected subcutaneously into nude mice	*In vivo*	Tan IIA:5,10 mg/kg/d, i.p. for 21days	↓miR-21-5p, ↑Occludin, ↑Occludin mRNA, ↑ZO1, ↑ZO1 mRNA in cells and the lung tissue	Zhu et al. (2023)
	A549 cells	*In vitro*	Tan IIA:4 μg/ml (13.6 μM) incubated for 24h and 48h		
	C57BL/6 mice [male, n = 48]; mice were subjected to intermittent hypoxia from 8:00 a.m. to 4:00 p.m. daily with 120 SEC cycles of hypoxia (6%–8%) and reoxygenation (21%) for 5 weeks. Lewis lung cancer was injected subcutaneously into the right abdomen of mice at week 1	*In vivo*	Tan IIA:10 mg/kg/d i.p for when the tumor volume reached about 200mm^3^, 5 weeks of modeling and therapy	↓MDA, ↑SOD, ↑BAX, ↑Cleaved caspase-3, ↑Nrf2, ↓p-NF-kB p65 in the lung tissue	Zhang et al. (2020)
	Lewis lung carcinoma (LLC) cell lines; incubated in an oxygen control incubator (1%,1%–21%,21% and 21%-1% O2 incubate for 5 min each) during the 24h period	*In vitro*	Tan IIA:10 μg/mL incubated for 24h	↑miR-138, ↓HIF-1α, ↓VEGF, ↑BAX, ↑Caspase-3, ↑TUNEL in cells and the lung tissue	Zhang et al. (2024b)
	C57BL/6J mice [male, n = 48]; IH-induce (the oxygen content changed from 21% to a minimum of 6%–8% in 120 s, 8 h a day, for 5 weeks) One week after IH exposure, mice were injected with LLC cells subcutaneously on the right side	*In vivo*	Tan IIA:10 mg/kg/d, i.p, for 5weeks, when LLC administration begins approximately 5–7 days after injection		
	NSCLC cell lines (A549, H596, H1299, Calu-1 and H460 cell lines)	*In vitro*	Tan IIA:5,10,15,20,30,60 μM incubated for 24h	↑GRP78, ↑CHOP, ↑DR5, ↓p-STAT3, ↓survivin, ↑Cleaved caspase-3, ↑Cleaved caspase-8, ↑Cleaved-PARP in cell	Kim et al. (2016)
	Drug resistant NSCLC cell lines of A549 and H358; H1975 and H1650 cell lines	*In vitro*	ATA:0.125,0.25,0.5,1,2 μM incubated for 48h	↓p70S6K, ↓p-p70S6K, ↓p-S6RP, ↑p53, ↑p21, ↓Survivin, ↓D3, ↓AURKA, ↓PLK1, ↓Cyclin B1, ↓EGFR, ↓MET in cells and the lung tissue	Huang et al. (2022b)
	nude mice [female, n = 18]; A549 cells were injected subcutaneously into nude mice	*In vivo*	ATA:25 mg/kg i.p. for every 3 days for 31 days, when the tumor volume reached about 100mm3		
	Human SCLC H1688 and H446 cell lines	*In vitro*	Tan IIA:0,1,2,4,6 μmol/L incubated for various time points (24,48 and 72 h)	↓PI3K, ↓p-Akt, ↑E-cadherin, ↓vimentin, ↓EMT in cells and the lung tissue	Jiang et al. (2024)
	BALB/nude mice [male, n = 10]; H1688 cells were injected subcutaneously into the right axilla	*In vivo*	Tan IIA:10 mg/kg, i.p. qod,11 injections in total		
	The human NSCLC cell line A549 and PC9, and the Human Lung Fibroblast (HLF) cell line; ADM (0,0.25,0.5,1,2,4 μM)cultures with or without for 48h	*In vitro*	Tan IIA:0,5,10,20,40,80 μM incubated for 48h	↑Bax, ↑Cleaved caspase-3, ↓VEGF, ↓VEGFR2, ↓Bcl-2, ↓Caspase-3, ↓p-Akt, ↓p-PI3K in A549 cell	Xie et al. (2016)
	C57BL/6J mice [male, n = 80]; Lewis tumor cells were inoculated subcutaneously into the right armpit of mice	*In vivo*	Tan IIA:15 mg/kg/d, i.p. for 2weeks, with or without CTX (25 mg/kg) i.p. qod	↑Bcl-2, ↑Bax, ↓VEGF, ↑angiostatin, ↑endostatin, ↑CD4+, ↑CD4+/CD8+ in the lung tissue	Li et al. (2016)
	The NSCLC cell lines A549, PC9, H1299, and SPA-A1	*In vitro*	Tan IIA:0,1.25,2.5,5,10,20,40 μM incubated for 24, 48 and 72h, respectively; with DDP (0,0.0625,0.125,0.25,0.5,1,2 μM)	↑Bax, ↑Cleaved caspase-3, ↓ p-PI3K, ↓p-Akt, ↓Caspase-3, ↓Bcl-2 in cell and the lung tissue	Liao et al. (2019)
	BALB/c-nu mice [/, n = 24]; A549 cells were inoculated subcutaneously into one axillary pit of mice	*In vivo*	Tan IIA:15 mg/kg, i.p. biw for 4weeks or Tan IIA (7.5 mg/kg) combined with DDP (1.5 mg/kg) i.p. biw for 4weeks, when the tumor volume reaches 300mm3		
	H1975, PC9, H1650, H2228, H460, LLC and BEAS-2B cells	*In vitro*	Tan IIA: 0,5,10,20,30,40 μmol/L incubated for 24h or 48h	↑p-JNK, ↓NFAT2, ↓c-Myc in cell and the lung tissue; ↑p-c-Jun-S73, ↑PD-L1 in H1975 and PC9	Zhang et al. (2024c)
	nude mice [female, n = 25]; H1975 cells were injected subcutaneously	*In vivo*	Tan IIA:10,20,40 mg/kg/d, i.p. for 18days, when the tumor volume reached about 50–100mm3		
	C57 mice [female, n = 25]; LLC cells were injected subcutaneously	*In vivo*	Tan IIA:15,30,60 mg/kg/d, i.p. for 18days, when the tumor volume reached about 50–100mm3		
			Tan IIA:40 mg/kg/d, i.p. for 15days with or without PD-1 mAb (10 mg/kg/q3d), when the tumor volume reached about 50–100mm3		
	The NSCLC cell lines HCC827 and PC-9	*In vitro*	Tan IIA:0,1,2,4,8,16 μM incubated for 72h or gefitinib (0,5,10,20,40,80,160,320 nM)	↓p-EGFR. ↓p-VEGFR2,↓p-Akt in HCC827 cell	Wang et al. (2019b)
			Tan IIA:2 μM combined with gefitinib (0,10,20,40,80,160,320 nM) incubated for 12h, then gefitinib (40 nM) or/and Tan IIA (2 μM) treat for 3d		
	BALB/c nude mice [male, n = 24]; HCC827/Gefitinib cells were inoculated subcutaneously into the right abdomen of mice	*In vivo*	Tan IIA:20 mg/kg/d combined with or without gefitinib (150 mg/kg/d) i.p. for 3weeks, when the tumor volume reached about 200mm3	↓p-VEGFR, ↓p-Akt in the lung-tissue	
	H1975 and H1975/OR cells	*In vitro*	Tan IIA:0.3125,0.625,1.25,2.5,5 μM incubated for 72h	↓SREBPs, ↓SREBP1, ↓FASN, ↓SCD, ↓SREBP2, ↓HMGCS1, ↑unsaturated lipids, ↓GSH, ↑ROS in cells and the lung tissue	Cao et al. (2024)
	BALB/c nude mice [female, n = 24]; H1975 cells OR H1975/OR cells were injected subcutaneously into the right abdomen of all mice	*In vivo*	Tan IIA:20 mg/kg^−1^/d, intragastric administration with or without osimertinib (5 mg⋅kg^−1^) for 4weeks, when solid tumor volume reached 50–100 mm^3^ administration		
	PC9, PC9/GR cells and H1975	*In vitro*	Tan IIA:2.5 μM with or without Gefitinib (PC9:0.1μM; PC9/GR:1 μM) incubated for 2weeks	↓ SREBP1, ↓FASN, ↓SCD, ↑ROS, ↑MDA in cell and the lung tissue	Zhang et al. (2024d)
	BALB/c nude mice [female, n = 24]; PC9/GR was injected subcutaneously into the right hypochondrium of mice	*In vivo*	Tan IIA:20 mg/kg/d with or without Gefitinib (25 mg/kg/d), oral tube feeding, when the tumor size reaches 100 mm^3^		

## 2 Methodology

In this study, the search strings “ (Tanshinone IIA)” and “ (lung)” were used to collect articles published from 2014 to June 2024 in PubMed and Web of Science. A total of 109 documents were retrieved, among which 87 were research articles and six were review articles. Notably, 46 articles focused on the potential mechanisms of Tan IIA in combating respiratory diseases. For articles with a pre - existing research foundation, relevant articles from previous studies were also included. Studies that did not consider Tan IIA and its derivatives as active substances were excluded. After a rigorous screening process, a total of 51 articles were finally included in this in - depth analysis. Based on the comprehensive analysis of the included literature, the research directions regarding the mechanisms of Tan IIA against respiratory diseases were clarified, and the basic structure of this paper was determined. Through the meticulous analysis of these selected articles, this study aims to provide valuable references for the clinical application of Tan IIA in the treatment of respiratory diseases.

## 3 Effect and mechanism of Tan IIA against respiratory diseases

### 3.1 Asthma

Asthma is a heterogeneous disease of the respiratory system, characterized by chronic airway inflammation and airway hyperreactivity. Its symptoms are mostly induced by exposure to allergens. The pathological changes during attacks include inflammatory cell infiltration in the bronchi, mucosal edema, glandular hypersecretion, and tracheal smooth muscle contractions. It is characterized by high morbidity and a low cure rate, though mortality is generally low with proper management. According to relevant epidemiological studies (Porsbjerg et al., 2023), more than 300 million people worldwide are estimated to have asthma, and with advancements in medicine, the incidence of asthma now seems to be stabilizing. Zhang et al. (Zhang et al., 2017) discovered that STS directly acts on mouse tracheal smooth muscle cells (ASMCs) to block L-type calcium channels, thereby reducing extracellular calcium influx and relaxing precontracted mouse tracheal rings. Their findings suggest that STS could be a promising treatment for asthma by activating ATP-sensitive potassium channels, hyperpolarizing the membrane, and further limiting calcium influx. Wang et al. (Wang S. B. et al., 2019) found that in a mouse model of ovalbumin (OVA)-induced asthma, Tan IIA may inhibit the increase of inflammatory cells in the blood by suppressing Th2 cytokine activity in lung tissue and inhibiting nuclear factor-κB (NF-κB) activation, while increasing heme oxygenase-1 (HO-1) activity. This results in decreased levels of Th2-associated cytokines (IL-13, IL-4, and IL-5) in bronchoalveolar lavage fluid (BALF). Additionally, Tan IIA significantly reduces acetylcholine-stimulated lower respiratory tract resistance and tissue elasticity, thus inhibiting airway hyperreactivity to some extent. Furthermore, Tan IIA upregulates the expression of antioxidant enzymes such as nuclear factor erythroid 2 (Nrf2), glutathione peroxidase (GPx), and superoxide dismutase (SOD) in lung tissue, while decreasing levels of malondialdehyde (MDA) and reactive oxygen species (ROS) to regulate the oxidant-antioxidant balance in asthma, thereby reducing oxidative stress. Heo et al. (Heo and Im, 2019) revealed through *in vitro* studies that Tan IIA inhibits anti-dinitrophenyl (DNP) IgE-induced expression of β-hexosaminidase in RBL-2H3 mast cells, thereby reducing FcεRI-mediated mast cell degranulation and exerting anti-allergic effects. *In vivo* studies further showed that Tan IIA suppresses Th2 cytokine production in lung tissue, reduces mRNA expression levels of IL-4, IL-5, IL-13, and interferon-γ (INF-γ) in BALF, decreases eosinophil accumulation in BALF, and significantly reduces endobronchial mucin production. Ultimately, these effects reduce lung inflammation and inhibit glandular secretion in OVA-induced asthma mouse models. In summary, Tan IIA can relax bronchial smooth muscle, reduce airway inflammation, and inhibit airway gland secretion, thus exhibiting anti-asthmatic activity and potentially serving as an effective alternative therapy for asthma treatment.

### 3.2 Chronic obstructive pulmonary disease

Chronic obstructive pulmonary disease (COPD) is a respiratory condition marked by persistent, irreversible airflow limitation due to chronic bronchitis and emphysema. Tobacco smoke is the primary risk factor, while infections often exacerbate symptoms. The prevalence and mortality of COPD have risen in recent years, making it the third leading cause of death globally in 2019 (Christenson et al., 2022). Studies have explored the potential of STS in COPD treatment. Chen et al. (Chen et al., 2017) found that STS ameliorates airway dehydration by activating calcium-activated chloride channels in tracheal epithelial cells, increasing intracellular Ca^2+^ via muscarinic acetylcholine receptors, and stimulating Cl−secretion. Li et al. (Li et al., 2018) demonstrated that STS inhibits CS/CSE-induced reductions in CFTR, thereby blocking ERK1/2 and NF-κB activation, reducing IL-6 and IL-8 secretion, and mitigating inflammation, airway remodeling, and epithelial hyperplasia. Guan et al. (Guan et al., 2018) showed that STS blocks MAPK signaling and reduces HIF-1α protein synthesis, inhibiting the HIF-1α pathway and downregulating TNF-α and IL-1β expression. It also reduces ROS, HO-1, and NOX1 production, thereby decreasing lung inflammation and oxidative stress. Further, STS may inhibit HIF-1α expression by reducing phosphorylation of ERK, p38 MAPK, and JNK in macrophages.

In another study, Li et al. (Li et al., 2020) found that STS significantly inhibits ERK1/2 phosphorylation and NF-κB p65 activity, reducing IL-6 and IL-8 release and reversing mucus hypersecretion and lung function decline in a mouse model of COPD acute exacerbation. Guan et al. (Guan et al., 2021) also noted that STS improves mitochondrial function by up-regulating SIRT1, increasing mitochondrial membrane potential, and enhancing mitochondrial respiratory chain complex activity, while reducing ROS and apoptosis. Overall, these studies suggest that STS has potential as a therapeutic agent for COPD by targeting multiple pathways involved in the disease process.

### 3.3 Pulmonary hypertension

Pulmonary hypertension (PH) is a multifactorial, progressive cardiopulmonary disease characterized by high morbidity and mortality. Among its various forms, chronic hypoxic pulmonary hypertension (CHPH) stands out as a leading cause of progressive cardiopulmonary disease. Hypoxia triggers pulmonary vasoconstriction and stimulates the proliferation of pulmonary artery smooth muscle cells (PASMC), leading to pulmonary vascular remodeling (PVR) and ultimately resulting in PH. If left untreated, PH can significantly alter the structure and function of the right heart, culminating in right heart failure (Rabinovitch et al., 2014). Huang et al. discovered that in a rat model of hypoxia-induced pulmonary hypertension (PH), Tan IIA could inhibit hypoxia-induced thickening of the pulmonary artery wall, significantly reduce the mean pulmonary artery pressure (mPAP) and the ratio of right ventricular weight to the sum of left ventricular and septal weights [RV/(LV + S)], and suppress the thickening of the pulmonary artery wall. Through *in vitro* experiments on hypoxia-induced pulmonary artery smooth muscle cells (PASMCs), it was discovered that the above effects might be related to Tan IIA upregulating the expression of Kv2.1 protein (Huang et al., 2009). This team further investigated and found (Zheng et al., 2015) that Tan IIA might restore oxygen-sensitive voltage-gated potassium channels by increasing the expression of Kv1.5 and Kv2.1, upregulate the voltage-gated potassium channel current (Ikv), inhibit voltage-dependent calcium channels (VDC), and reduce the intracellular free calcium ion concentration (Ca^2+^) Cyt, thereby inhibiting hypoxia-induced proliferation of PASMCs and exerting a therapeutic effect on PH. Wang et al. (Wang et al., 2010) applied different doses of Tan IIA to PASMCs of rats with hypoxia-induced pulmonary hypertension *in vitro*. It was detected that a low concentration of Tan IIA could enhance hypoxia-induced initial contraction of the pulmonary artery, while a high concentration of Tan IIA had the opposite effect. Meanwhile, it was discovered that Tan IIA might mainly induce pulmonary artery vasodilation by inhibiting the influx of extracellular Ca^2+^, partly by inhibiting the release of intracellular Ca^2+^ and the activation of Ca^2+^-activated K^+^ channels (K_Ca_ channels), and thus had the potential to treat hypoxic pulmonary hypertension. Wang et al. (Wang et al., 2013a) found that STS significantly inhibited CHPH and monocrotaline (MCT)-induced expression of transient receptor potential channel TRPC1 and transient receptor potential cation channel 6 (TRPC6) in distal pulmonary arteries, pulmonary vascular smooth muscle, and PASMC in SD rats. This reduction in expression decreased calcium pool operant calcium influx (SOCE) and intracellular Ca^2+^ increases, thereby regulating Ca^2+^ homeostasis in PASMC, inhibiting their proliferation and migration, and reducing pulmonary vascular resistance and remodeling. Furthermore, STS was able to reduce hypoxia-induced mean pulmonary arterial pressure (MPAP), right ventricular systolic pressure (RVSP), and the right ventricular/left ventricular plus interventricular septum weight ratio [RV/(LV + S)%], preventing the progression of PH induced by hypoxia. The team further discovered that Tan IIA acts against PH by up-regulating the expression levels of protein kinase G (PKG) and peroxisome proliferator-activated receptor γ (PPAR-γ) in pulmonary vascular smooth muscle and hypoxia-induced rat PASMCs in a hypoxia-induced rat PH model. This upregulation repairs the PKG-PPAR-γ signaling axis, reduces the expression levels of transient receptor potential canonical protein (TRPC), and inhibits the SOCE enhancement of hypoxia-induced PASMC (Jiang et al., 2016). Luo et al. discovered (Luo et al., 2013) that Tan IIA might regulate the Akt/Skp2-related pathway by reversing the increase in S-phase kinase-associated protein 2 (Skp2) and the phosphorylation of Akt, and then upregulate the protein level of P27. It could arrest PASMCs in the G1/G0 phase, inhibit hypoxia-induced proliferation of PASMCs, and ultimately attenuate pulmonary artery remodeling. Bao et al. (Bao et al., 2020) found that STS could reduce the expression levels of phosphatidylinositol 3 (PI3), phosphorylated Akt (p-Akt), mammalian target of rapamycin (mTOR), phosphorylated mTOR, S6 kinase (S6K1), and phosphorylated S6K1 in hypoxia-induced lung tissues of SD rats. This reduction inhibited the PI3K/AKT/mTOR pathway, upregulated autophagy levels in lung tissue cells, and exerted anti-PH effects. In addition, STS decreased the expression of proinflammatory cytokines IL-6, IL-8, and TNF-α in the lung tissue of hypoxia-induced SD rats, thereby reducing pulmonary inflammation. Wang et al. (Wang et al., 2022) found that STS could decrease RVSP, Fulton index [FI, RV/(LV + S)], increase cardiac output (CO), and inhibit the production of α-smooth muscle actin (α-SMA), von Willebrand factor (VWF), apoptotic marker Bax, and cleaved cysteine aspartate aminotransferase 3 (Caspase 3) in pulmonary vascular endothelial cells. This inhibition prevented pulmonary vascular endothelial cell apoptosis and the development of CHPH in rats. Further studies revealed that STS could upregulate bone morphogenetic protein type II receptor (BMPR2) expression and enhance BMP9-induced phosphorylation of Smad1/5/9 in hypoxia-induced rat pulmonary microvascular endothelial cells (PMVECs) and human embryonic stem cell-derived endothelial cells (HESC-ECs). This enhancement of BMP9-BMPR2-Smad1/5/9 signaling exerted anti-apoptotic effects.

### 3.4 Pulmonary fibrosis

Pulmonary fibrosis (PF) is a chronic, progressive, fibrosing interstitial pneumonia that ultimately often progresses to respiratory failure or even death, with a high mortality rate. Pathological changes primarily occur in the pulmonary interstitium and are characterized by the infiltration of the alveolar wall by various combinations of inflammatory cells, fibrosis, and the proliferation of certain cells that constitute the normal alveolar wall (Wijsenbeek and Cottin, 2020). Wu et al. (Wu et al., 2014) reported that Tan IIA enhances ACE-2 and Ang- (1–7) levels in BLM-treated rat lung tissue, modulating the ACE-2/Ang- (1–7) axis. This regulation suppresses TGF-β1 expression, thereby attenuating collagen accumulation and fibrosis. He et al. (He et al., 2015) found that Tan IIA reduces the expression levels of tumor necrosis factor-β (TNF-β), IL-1β, and IL-6 in the bronchoalveolar lavage fluid (BALF) of BLM-induced rats and also inhibits the expression of inducible nitric oxide synthase (iNOS) in lung tissue, thus reducing NO production and alleviating the inflammatory response in pulmonary fibrosis. In addition, Tan IIA also inhibits cyclooxygenase-2 (COX-2)-related oxidation in BLM-induced rat lung tissue, reduces the expression levels of COX-2, prostaglandin E2 (PGE2), and malondialdehyde (MDA) in lung tissue, and exerts antioxidant effects. The team further found that Tan IIA inhibits the phosphorylation of Smad-2/3 and reduces collagen I production in BLM-induced rats and in human alveolar epithelial cell A549 induced by recombinant transforming growth factor-β1 (TGF-β1), thereby blocking TGF-β signaling, inhibiting the expression levels of α-smooth muscle actin (α-SMA), fibronectin (FN), and vimentin, and reducing epithelial-mesenchymal transition (EMT). Tan IIA also reduces the infiltration of CD-68-positive macrophages in BLM-induced rat lung tissue and decreases lung tissue inflammation (Tang et al., 2015). Feng et al. (Feng et al., 2019) found that Tan IIA reduces the levels of tumor necrosis factor-α (TNF-α), IL-6, and IL-1β, inhibits the expression of type I collagen, FN, and α-SMA, and decreases the expression levels of TGF-β1, p-Smad2/3, and p-Smad3 while up-regulating the expression of Smad7 in the lung tissue of a rat model of silica-induced pulmonary fibrosis. Tan IIA also inhibits the expression of nicotinamide adenine dinucleotide phosphate oxidase 4 (NOX4) in lung tissue, upregulates the mRNA expression levels of nuclear transcription factor erythroid 2-related factor 2 (NRF2) and its downstream proteins heme oxygenase 1 (HO-1) and NAD (P)H quinone dehydrogenase 1 (NQO-1) in lung tissue, which in turn reduces the production of ROS and MDA and increases the activities of superoxide dismutase (SOD) and glutathione peroxidase (GSH-Px). This suggests that Tan IIA inhibits the activation of the TGF-β1/Smad1/Smad3 signaling pathway and activates the Nrf2/antioxidant response element (ARE) signaling pathway, thereby reducing pulmonary inflammation and oxidative stress and slowing pulmonary fibrosis and lung injury. The team further found that Tan IIA inhibits SiO2-induced expression of TGF-β1 and its downstream p-Smad3 in human lung adenocarcinoma cell line (A549) and human bronchial epithelial cell (HBE) cells, upregulates the expression level of Smad7, and increases the expression of Nrf2, HO-1, and NQO-1, thereby inhibiting the TGF-β1/Smad signaling pathway and EMT (Feng et al., 2020). Jiang et al. (Jiang et al., 2020) found that STS downregulates endotoxin-induced IL-1β and TNF-α expression levels in THP-1 macrophages, reduces inflammatory responses, and inhibits human embryonic lung cell (MRC-5) proliferation and fibroblast-to-myofibroblast transformation (FMT) in co-culture. In addition, STS inhibits TGF-β1-induced overexpression of collagen type I α1 (COL1α1) and α-SMA in MRC-5 cells, thereby inhibiting FMT, which in turn inhibits cell proliferation and exerts anti-fibrotic effects. Zhu et al. (Zhu et al., 2020) found that STS inhibits the expression of type I and type III collagen, downregulates hydroxyproline levels, reduces ROS and MDA levels, and protects cellular lipids from peroxidation in the alveolar space of rats with SiO2-induced pulmonary fibrosis, thereby reducing the degree of pulmonary fibrosis. It was further found that STS may upregulate Nrf2 expression and nuclear translocation, increase thioredoxin (Trx) and thioredoxin reductase (TrxR) transcription, and activate the Nrf2/Trx/TrxR axis in lung tissue of SiO2-induced fibrotic rats, thus exerting an anti-fibrotic effect. Zhang et al. (Zhang H. et al., 2024) found that Tan IIA downregulates the expression levels of zinc finger and BTB domain 16 (Zbtb16), downregulates the expression of type I collagen, fibronectin, and α-SMA, reduces the phosphorylation of Smad2/3, inhibits the activation of the TGF-β1/Smad signaling pathway, inhibits the level of hydroxyproline (HYP) in lung tissue, and reduces the lung index (lung weight/body weight) in the BLM-induced rat pulmonary fibrosis model and the TGF-β1-treated MRC-5 model, thereby exerting its effect against pulmonary fibrosis.

### 3.5 Acute lung injury/respiratory distress syndrome (ALI/ARDS)

Acute lung injury (ALI) is a prevalent and severe lung condition that arises from damage to alveolar epithelial and capillary endothelial cells, triggered by various intrapulmonary or extrapulmonary factors. In its most severe form, ALI can progress to acute respiratory distress syndrome (ARDS) (Bos and Ware, 2022). According to the 2012 Berlin definition, ALI is uniformly classified as ARDS based on the PaO2/FiO2 ratio (mild≤300 mmHg, moderate≤200 mmHg, severe≤100 mmHg). Common risk factors for ALI/ARDS include sepsis, trauma, massive blood transfusion, pneumonia, and inhalation injury, with sepsis being the leading cause of mortality (Bos and Ware, 2022). The cytology of ALI/ARDS is characterized by the disruption of alveolar-capillary membrane integrity, excessive transepithelial migration of neutrophils, activation of inflammatory and oxidative stress factors, increased vascular permeability, and histologically by features such as pneumonitis, pulmonary edema, hyaline membrane formation, and diffuse alveolar damage (Long et al., 2022). Epidemiological studies suggest that ALI/ARDS affects roughly three million patients worldwide annually, accounting for approximately 10% of intensive care unit (ICU) patients and 24% of ICU patients requiring mechanical lung ventilation, which remains an effective treatment for this condition (Fan et al., 2018; Bellani et al., 2016; Fan et al., 2005).

In sepsis-induced ALI/ARDS, Xu et al. detected (Xu et al., 2009) that in a mouse model of acute lung injury induced by lipopolysaccharide (LPS), Tan IIA might relieve pulmonary edema, reduce the destruction of lung structures, and exert a protective effect on LPS-induced lung injury by inhibiting the activation of NF-κB, further reducing the activity of phospholipase A2 (PLA2), downregulating the activity of myeloperoxidase (MPO) in lung tissues, and decreasing the protein content and the number of neutrophils in bronchoalveolar lavage fluid. This effect was confirmed by *in vitro* cell experiments. This team further investigated and found (Xu et al., 2011) that Tan IIA might reduce the expression of hypoxia-inducible factor 1 subunit α (HIF-1α) in a mouse lung injury model induced by LPS and in macrophages by inhibiting the PI3K/AKT and MAPK pathways as well as relevant protein translation regulatory factors. Moreover, Tan IIA could promote the degradation of HIF-1α protein through the proteasome pathway in LPS-stimulated macrophages, thereby downregulating the expression levels of TNF-α, IL-1β, and IL-6, and ultimately alleviating the degree of LPS-induced lung injury. Wang et al. (Wang et al., 2018) found that Tan IIA reverses the decreased expression of ACE2 and Ang- (1–7) in lung tissue of a paraquat (PQ)-induced rat model, activates the ACE2/Ang- (1–7) axis, and reduces neutrophil count, lactate dehydrogenase (LDH) content, myeloperoxidase (MPO) activity, and the wet/dry weight (W/D) ratio in lung tissue, exerting a therapeutic effect. Quan et al. (Quan et al., 2019) reported that Tan IIA upregulates the level of silent information regulator (Sirt1), decreases NF-kB p65 activity, and regulates the Sirt1/NF-kB signaling pathway, thereby down-regulating TNF-α, IL-1β, and IL-6 levels, reducing MPO activity, decreasing the W/D ratio of lung tissue, and improving the damaged alveolar structure and exudative pulmonary edema in lipopolysaccharide-induced mouse lung tissue. Zhao et al. (Zhao et al., 2022) observed that Tan IIA reduces inflammatory factors such as IL-6, IL-8, and TNF-α in LPS-induced mouse lung tissue, reverses lipopolysaccharide-induced alveolar wall thickening, alveolar epithelial injury, and lymphocyte aggregation. Further *in vitro* studies revealed that Tan IIA regulates LPS-induced polarization of mouse macrophages (MMAs), increases the relative amount of M2 subtype, and decreases the relative amount of M1 subtype, thereby inhibiting NF-kB p65 and hypoxia-inducible factor 1 subunit α (HIF1α) expression, reducing inflammation, and promoting the repair of mouse alveolar epithelial cells (MAECs) in LPS-induced injury. Liu et al. (Liu et al., 2022) found that Tan IIA inhibits the ROCK2/NF-κB axis by suppressing ROCK2 expression in septic rats induced by cecal ligation and puncture (CLP) and lipopolysaccharide-treated RLE-6TN cells, down-regulating the expression of Bax and cleaved caspase-3 protein, and decreasing IL-1β, TNF-α, and IL-6 levels, thus alleviating lung tissue and cell injury and exerting anti-ALI/ARDS effects.

Regarding trauma-induced ALI/ARDS, Liu et al. (Liu et al., 2020) reported that Tan IIA reverses the low expression of B-cell lymphoma 2 (Bcl2) and the high expression of Bax and caspase-3 after lung impact injury by down-regulating phosphorylated phosphatidylinositol-3-kinase (p-PI3K) and phosphorylated protein kinase B (p-Akt), up-regulating Forkhead transcription factor phosphorylation (p-FoxO1), and regulating the PI3K/Akt/FoxO1 signaling pathway in a shockwave-induced acute lung injury mouse model. This reduces the expression of reactive oxygen species (ROS), MDA5, and inositol-requiring kinase 1α (IRE-α), increases superoxide dismutase 1 (SOD-1) production, inhibits the expression of TNF-α, IL-6, and IL-1β, and increases IL-10 expression, thereby reducing apoptosis, oxidative stress, and the inflammatory response in lung tissue cells and protecting against acute lung injury caused by lung impact. Tai et al. (Tai et al., 2021) found that Tan IIA combined with cyclosporine A (CsA) improves mitochondrial function by up-regulating PI3K, p-Akt, p-Bad, and Bcl-2 expression, down-regulating Bax, Cyt-c, caspase-3, and PARP expression, and activating the PI3K/Akt/Bad signaling pathway in an ALI model induced by renal ischemia-reperfusion injury (IRI) in obese rats. This combination also increases mitochondrial DRp1 expression, decreases Mfn1 and Mfn2 expression, significantly increases PGC-1α, NRF1, and TFAM mRNA and protein levels, improves mitochondrial biogenesis and kinetic abnormalities, reduces ROS levels and MPTP opening, increases mitochondrial RCR and ATP levels, increases the ratio of long/short segments, and reduces mitochondrial DNA damage. These effects suggest that Tan IIA reduces lung tissue apoptosis, lung injury, and improves lung function in IRI-induced obese rats primarily by regulating mitochondrial function. Zhang et al. (Zhang et al., 2023) reported that Tan IIA increases the phosphorylation of PI3K, Akt, and mammalian target of rapamycin (mTOR) in lung tissue, activating the PI3K/Akt/mTOR signaling pathway. This upregulates Bcl2 expression, decreases Bax, Bim, Bad, and Caspase3 expression, reduces the number of TUNEL-positive cells, and upregulates glutathione peroxidase 4 (Gpx4), cystine/glutamate reverse transporter (SLC7A11), and glutathione (GSH) levels in lung tissue. Tan IIA also decreases the expression of prostaglandin endoperoxidase 2 (Ptgs2) and malondialdehyde (MDA), inhibits apoptosis and ferroptosis, reduces the expression of TNF-α, IL-6, and IL-1β, increases IL-10 expression, decreases the W/D ratio and MPO content, and increases SOD content, thereby alleviating I/R-induced acute lung injury.

### 3.6 Lung cancer

Lung cancer is a prevalent malignancy with notably high mortality rates. In 2018, it accounted for approximately 18.4% of total cancer deaths, resulting in 1.8 million fatalities worldwide. Histopathologically, lung cancer is divided into small cell lung cancer (SCLC) and non-small cell lung cancer (NSCLC), with NSCLC being the more prevalent, representing over 85% of cases. Adenocarcinoma and squamous cell carcinoma constitute 40% and 25% of NSCLC cases, respectively. In terms of genetic driver mutations, the EGFR subtype pattern is the most prevalent (Nicholson et al., 2022; Schabath and Cote, 2019). According to recent studies, smoking remains the paramount risk factor for lung cancer (Leiter et al., 2023). For patients in the early stages, surgical intervention is optimal. However, for those in the middle and advanced stages, chemotherapy, targeted therapy, radiotherapy, and immunotherapy are primarily utilized, albeit with limitations such as tumor cell drug resistance (Schuler et al., 2023).

Regarding the efficacy of Tan IIA monotherapy on lung cancer, particularly NSCLC, Xie et al. (2015) discovered that Tan IIA inhibits VEGFR2 activity by binding to the kinase domain of VEGFR2 protein in NSCLC cell line A549 cells. This downregulates VEGFR2 expression, blocks VEGF and VEGFR2 binding, decreases VEGF expression, and inhibits the VEGF/VEGFR signaling pathway. Consequently, it arrests the A549 cell cycle in the S phase and induces tumor cell apoptosis, exhibiting anti-tumor growth effects. Gao et al. (2020) found that Tan IIA downregulates downstream Akt and ERK1/2 activities, enhances endogenous Mcl-1 ubiquitination, promotes Mcl-1 degradation, and regulates the EGFR/Akt/Mcl-1 signaling pathway. This subsequently inhibits cell viability, proliferation, and induces apoptosis by decreasing the phosphorylation of wild-type (A549) and activating mutant (HCC827 and H1975) EGFR in NSCLC cells. This effect was validated in the HCC827 xenograft mouse model. Li et al. (2021) utilized bioinformatics analysis, network pharmacology, and molecular docking to simulate the potential mechanism of Tan IIA in treating lung adenocarcinoma. They identified 10 keys differentially expressed genes (DEGs) and found that the CCNA2-CDK2 complex had the strongest binding to Tan IIA. Further cellular studies revealed that Tan IIA downregulates the expression levels of CCNA2, CDK2, AURKA, PLK1, and ERK, inhibits the production of the CCNA2-CDK2 complex, and inhibits the AURKA/PLK1 pathway in A549 and H1975 cells. This results in the inhibition of cell proliferation, induction of cell cycle arrest in G1 and S phases for A549 and H1975 cells, respectively, and promotion of apoptosis. Sun et al. (2021) discovered that Tan IIA activates the PERK/ATF4/CHOP signaling pathway by up-regulating p-PERK, ATF4, and CHOP levels on the cell surface of NSCLC cell lines A549, H460, and H1299. This significantly upregulates the expression of ULBP1 and DR5 by activating ER stress signaling. Tan IIA also upregulates CD107α expression on the surface of NK cells stimulated by NSCLC cells and promotes NK cell degranulation. This enhances NK cell-target cell recognition and bolsters the immune surveillance and killing effect of NK cells on NSCLC. *In vivo* studies confirmed that Tan IIA, combined with NK cells, significantly inhibits tumor growth and enhances NK cell killing of NSCLC cells in SCID-BG mice subcutaneously transplanted with H460 cell xenografts. In C57BL/6 mice subcutaneously transplanted with LLC cells, Tan IIA significantly inhibited Lewis lung carcinoma (LLC) growth, and this effect was positively correlated with the number of NK cells. Qi et al. (2022) found that Tan IIA inhibits the expression of SIX1 and downregulates muscle pyruvate kinase isoenzyme 2 (PKM2), hexokinase (HK2), and lactate dehydrogenase (LDHA) levels in NSCLC cells both *in vitro* and *in vivo*. This reduces cellular glucose uptake, ATP levels, and lactate production, inhibits glycolysis, exerts an anti-Warburg effect, and subsequently inhibits the proliferation of NSCLC cells. Sun et al. (2023) discovered that Tan IIA downregulates the expression levels of CIRC0020123 and HMGB3, upregulates miR-1299 expression, and regulates the CIRC0020123/miR-1299/HMGB3 axis in NSCLC cells (A549 and H292). This subsequently inhibits proliferation, migration, invasion, and induces the apoptosis of NSCLC cells. Wang et al. (2023a) found that STS attenuates LUAD cell viability, migration, invasion, and angiogenesis by up-regulating miR-874 expression in NSCLC cells (A549 and H1299). This targets levels of eukaryotic elongation factor 2 kinase (eEF-2K), thereby inhibiting transglutaminase 2 (TG2) expression and regulating the miR-874/eEF-2K/TG2 axis. Zhu et al. (2023) discovered that Tan IIA decreases the expression level of miR-21-5p, promotes the expression of occludin and ZO1 protein and related mRNA in A549 xenograft mice and A549 cells *in vitro*. This subsequently inhibits the proliferation of A549 tumor cells, promotes apoptosis, and slows down the onset and progression of lung cancer. For NSCLC under hypoxia, Zhang et al. (2020) found that STS may reverse the decrease of BAX, cleaved caspase-3, and Nrf2, attenuate the increase of p-NF-kB p65, and mitigate IH-induced oxidative stress. This is achieved by decreasing MDA levels and increasing SOD levels in lung cancer cell tissues of a mouse model of intermittent hypoxia Lewis lung cancer. Zhang et al. (Zhang L. et al., 2024) discovered that STS attenuates IH-mediated tumor proliferation, migration, invasion, MVD, and increases tumor cell apoptosis. This is accomplished by up-regulating miR-138 expression, decreasing HIF-1α and VEGF levels, and increasing BAX, Caspase-3, and TUNEL-positive cell numbers levels in lung tissue and IH-induced Lewis lung carcinoma xenografted mice with obstructive sleep apnea (OSA) mimicking intermittent hypoxia (IH).For resistant NSCLC, Kim et al. (2016) found that Tan IIA activates the PERK/ATF4 pathway and increases CHOP by enhancing GRP78 activity in NSCLC cell lines. This induces DR5 upregulation while inhibiting STAT3 phosphorylation and down-regulating surviving in NSCLC cells. This subsequently upregulates cleaved-caspase-3, -8, and cleaved-PARP levels, increases TRAIL sensitivity in NSCLC cells, and induces TRAIL-resistant NSCLC apoptosis. Therefore, Tan IIA is a TRAIL sensitizer in NSCLC and holds potential for combination therapy with TRAIL. Huang et al. (Huang B. et al., 2022) discovered that acetyl tanshinone IIA (ATA) increases its ubiquitination by binding to p70s6k in drug-resistant NSCLC cells. This downregulates the expression levels of p70s6K, p-p70s6K, and p-s6RP in cells both *in vitro* and *in vivo*. Consequently, it increases p53 and p21 levels in cells, decreases the expression of cell cycle-related proteins (e.g., surviving, cyclin D3, AURKA, PLK1, cyclin B1) and receptor proteins (e.g., EGFR, MET), and blocks the cell cycle in the G1/S phase. This results in potent inhibition of growth, migration, and invasion of NSCLC cells with primary or acquired resistance to EGFR TKIs. For SCLC, Jiang et al. (2024) found that Tan IIA inhibits the PI3K/Akt signaling pathway by down-regulating the expression of PI3K and p-Akt. This increases E-cadherin expression, reduces vimentin expression, decreases EMT, and inhibits the proliferation and metastasis of SCLC cells in SCLC cell lines (H1688 and H446) and in an *in vivo* H1688 xenograft tumor model in mice.

**FIGURE 2:**
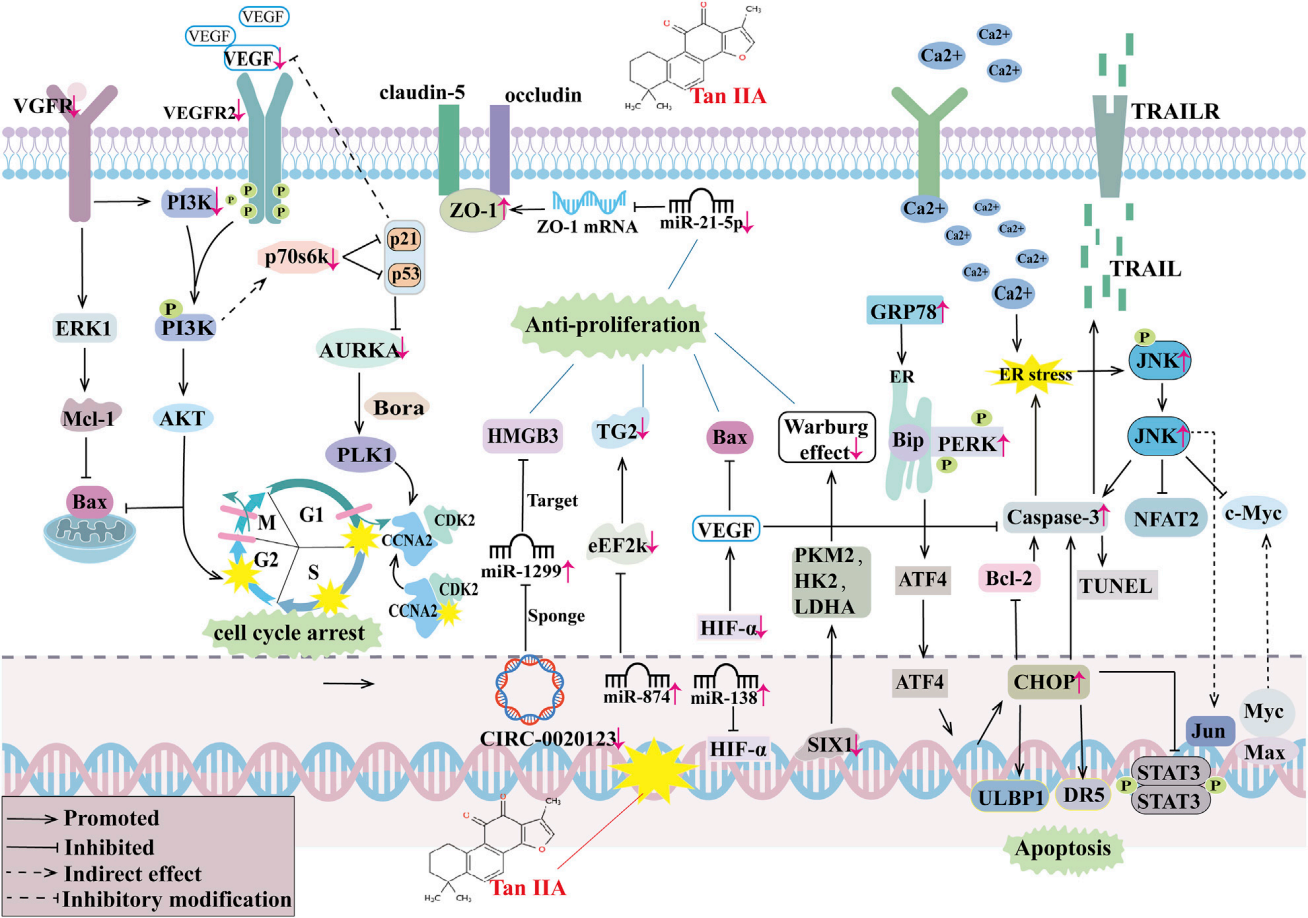
The antitumor mechanism of Tan IIA. Vascular growth factor receptor (VGFR), extracellular regulated protein kinases 1 (ERK1), Myeloid cell leukemia-1 (MCL-1), Bcl-2-associated X protein (BAX), Phosphoinositide 3-kinase (PI3K), protein kinase B (AKT), vascular endothelial growth factor (VEGF), vascular endothelial growth factor receptor 2(VEGFR2), The Rps6kb1 gene encodes a 70-kda ribosomal protein S6 kinase (P70s6k), aurora kinase A (AURKA), Activator of the protein kinase AURKA (Bora), Polo-like kinase 1 (PLK1), Cyclin-dependent kinase 2 (CDK2), cyclin A2(CCNA2), Zona Occludens 1 (ZO-1), high mobility group protein B3 (HMGB3), transglutaminase 2 (TG2), eukaryotic elongation factor 2 kinase (eEF-2K), hypoxia-inducible factor-α(HIF-α), sine oculis homeobox 1 (SIX1), muscle pyruvate kinase isoenzyme 2 (PKM2), hexokinase (HK2), lactate dehydrogenase (LDHA), endoplasmic reticulum (ER), glucose-regulated protein 78 (GRP78), heavy-chain binding protein (Bip), strand RNA-activated protein kinase-like ER kinase (PERK), activating transcription factor 4 (ATF4), cleaved cysteine aspartate aminotransferase 3 (Caspase 3), B lymphocytoma-2 gene (Bcl-2), C/EBP homologous protein (CHOP), UL16 Binding Protein 1(ULBP1), death receptor 5 (DR5), intracellular signal transducer and activator of transcription 3 (STAT3), Deoxynucleotide terminal transferase mediated dUTP Nick end labeling (TUNEL), Protoc gene-oncogene c-Jun (Jun), c-Jun N-terminal kinase (JNK), Nuclear Factors of activated T cells 2 (NFAT2), Myelocytomatosis viral oncogene homolog (Myc), MYC associated factor X (Max), tumor necrosis factor-related apoptosis-inducing ligand (TRAIL), TRAIL receptors (TRAILR).

When Tan IIA is combined with anti-tumor drugs, Xie et al. (2016) found that it enhances the efficacy of azithromycin in inhibiting the growth, reducing cell viability, and suppressing cell proliferation of A549, PC9, and HLF cells. Further studies revealed that the combination upregulates the expression levels of Bax and cleaved caspase-3, downregulates the expression levels of VEGF, VEGFR2, Bcl-2, caspase-3, p-Akt, and p-PI3K in A549 cells, and inhibits the VEGF/PI3K/Akt signaling pathway. This induces mitochondrial dysfunction, inhibits A549 cell migration and invasion, arrests cells in the G2 phase, and subsequently causes apoptosis. Li et al. (2016) discovered that the combination of Tan IIA and cyclophosphamide (CTX) significantly downregulates the expression of Bcl-2, upregulates the expression of Bax, and promotes the apoptosis of lung cancer cells in a Lewis tumor cell-induced mouse lung cancer model. This combination also decreases VEGF expression, increases the expression of angiostatin and endostatin, thereby inhibiting tumor tissue neovascularization. Liao et al. (2019) found that Tan IIA combined with cisplatin (DDP) may inhibit the PI3K/Akt signaling pathway by up-regulating Bax and cleaved caspase-3 expression levels and down-regulating p-PI3K, p-Akt, caspase-3, and Bcl-2 expression levels in NSCLC cells, thereby enhancing the anticancer ability of DDP in NSCLC and inhibiting NSCLC cell proliferation, migration and invasion. *In vivo* studies further confirmed that the combination of Tan IIA and DDP significantly inhibited tumor growth in A549-induced xenograft mouse lung cancer models compared with the Tan IIA and DDP monotherapy groups and could effectively enhance the antitumor effect of DDP. Zhang et al. (2024a) found that Tan IIA induced ER stress by increasing cytoplasmic Ca^2+^ levels, upregulating p-JNK levels, activating downstream JNK pathways, and decreasing NFAT2 and c-Myc expression in the cytoplasm and nucleus to regulate the JNK/NFAT2/c-Myc pathway and inhibit NSCLC growth in H1975 and PC9 xenograft tumor models and Lewis lung carcinoma models; meanwhile, Tan IIA may also enhance the expression of p-c-Jun-S73, upregulate membrane PD-L1 expression levels, and enhance the effect of anti-PD-1 immunotherapy for NSCLC by regulating JNK/c-Jun signaling in H197 5 and PC9 cells, which was confirmed in Lewis lung carcinoma models. For combination therapy with resistant cells, Tan IIA was able to improve the sensitivity of resistant cancer cells to resistant drugs. Wang et al. (2019a) found that Tan IIA combined with gefitinib could significantly inhibit the viability, proliferation, migration, and invasion of gefitinib-resistant HCC827 and PC-9 cells, and induce massive apoptosis of gefitinib-resistant NSCLC cells. Further studies revealed that Tan IIA combined with gefitinib could significantly downregulate the levels of p-EGFR, p-VEGFR2, and p-Akt, downregulate the VEGFR/Akt pathway, and enhance the sensitivity of gefitinib-resistant HCC827 to gefitinib in gefitinib-resistant HCC827 cells, thereby inhibiting the growth of HCC827 cells. *In vivo* studies also confirmed that Tan IIA combined with gefitinib inhibited the growth of xenografts by downregulating the VEGFR/Akt pathway in lung cancer tissues of gefitinib-resistant HCC827-induced xenografted mice. Cao et al. (2024) found that Tan IIA combined with osimertinib inhibited sterol regulatory element-binding proteins (SREBPs) signaling, downregulated the expression of SREBP1, fatty acid synthase (FASN), stearoyl-CoA desaturase (SCD), SREBP2, HMGCS1, inhibited SREBP signaling-mediated lipid desaturation and cholesterol synthesis, increased unsaturated lipid levels, decreased glutathione (GSH) levels, and increased ROS levels in osimertinib-resistant H1975/OR cells and reduced cell membrane fluidity, and reversed osimertinib-resistant H1975/OR cells’ resistance to osimertinib in both osimertinib-resistant H1975/OR cell and H1975/OR cell xenograft BALB/c mouse models. Zhang et al. (2024b) found that Tan IIA combined with gefitinib inhibited SREBP1-mediated *de novo* adipogenesis, increased ROS and MDA production, and increased the sensitivity of EGFR-mutant lung cancer resistant cells to gefitinib by down-regulating gene and protein expression of SREBP1, FASN, and SCD in lung cancer tissues of the drug-resistant cell lines PC9/GR, H1975, and PC9/GR human lung cancer cell xenograft mouse models, thereby inhibiting cell proliferation and promoting apoptosis. Therefore, we summarized the anti-tumor mechanism of Tan IIA, as shown in Figure 2.

## 4 Conclusion and prospects

**FIGURE 3:**
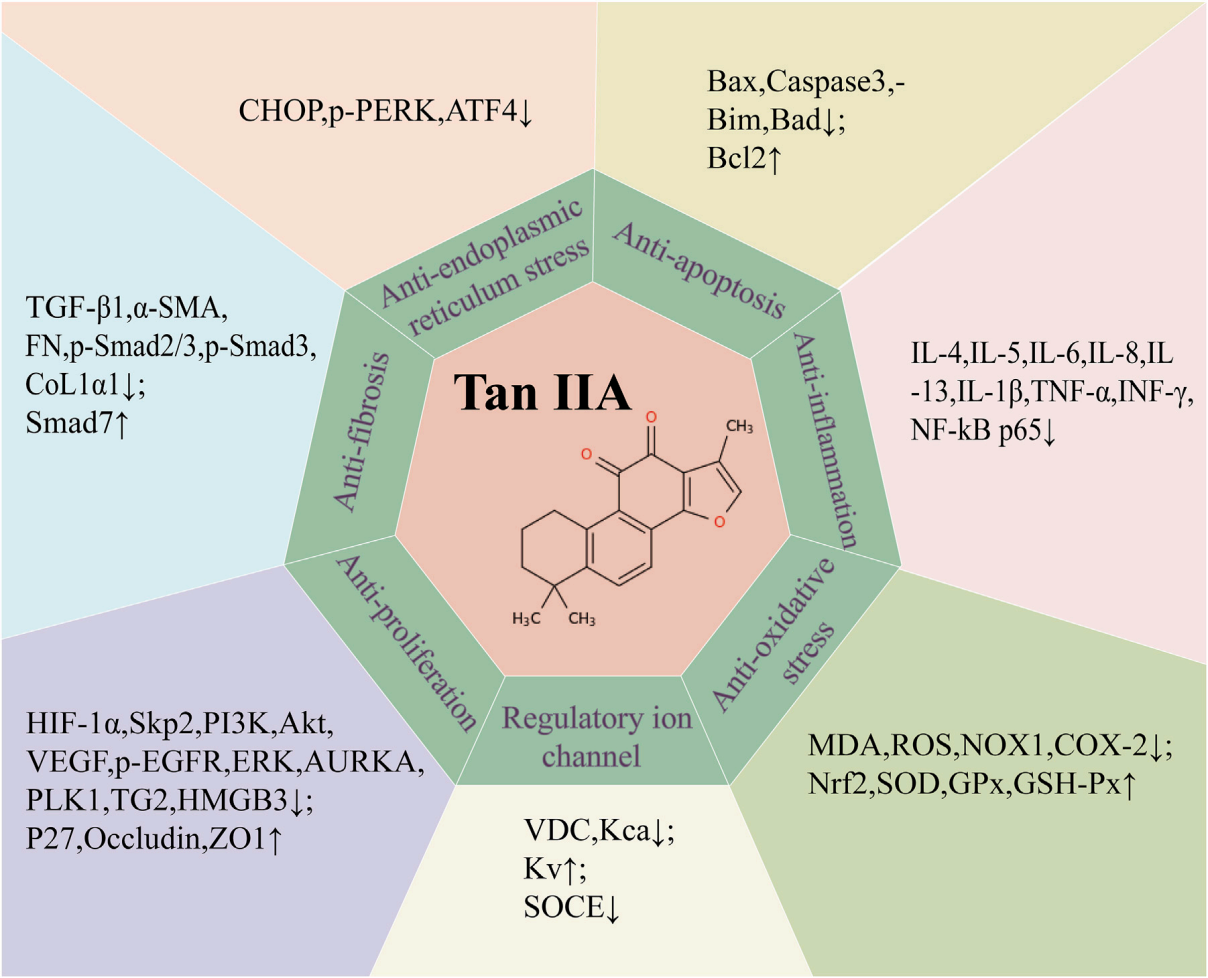
Summary of potential mechanism targets of Tan IIA against respiratory diseases. transforming growth factor-β1 (TGF-β1), α-smooth muscle actin (α-SMA), fibronectin (FN), collagen type I α1 (COL1α1), Bcl-2-associated X protein (BAX), cleaved cysteine aspartate aminotransferase 3 (Caspase 3), Bcl-2 interacting mediator of cell death (Bim), Bcl-xL/Bcl-2asociated death promoter (Bad), B lymphocytoma-2 gene (Bcl-2), Interleukin 4(IL-4), Interleukin 5 (IL-5), Interleukin 6 (IL-6), Interleukin 8 (IL-8), Interleukin 13 (IL-13), interleukin 1-β(IL-1β), tumor necrosis factor-α (TNF-α), interferon-γ (INF-γ), nuclear factor-κB (NF-κB), malondialdehyde (MDA), reactive oxygen species (ROS), NADPH oxidase 1(NOX1), cyclooxygenase-2(COX-2), nuclear factor erythroid 2 (Nrf2), superoxide dismutase (SOD), glutathione peroxidase (GPx), glutathion peroxidase (GSH-Px), C/EBP homologous protein (CHOP), protein kinase R-like endoplasmic reticulum kinase (PERK), activating transcription factor 4 (ATF4), hypoxia-inducible factor-α (HIF-α), S-phase kinase-associated protein 2 (Skp2), Phosphoinositide 3-kinase (PI3K), protein kinase B (Akt), vascular endothelial growth factor (VEGF), Phosphorylated epidermal growth factor receptor (p-EGFR), extracellular regulated protein kinases (ERK), aurora kinase A (AURKA), Polo-like kinase 1 (PLK1), transglutaminase 2 (TG2), high mobility group protein B3 (HMGB3),Cyclin-dependent kinase inhibitors (P27), Zona Occludens 1 (ZO1), Voltage dependent calcium channel (VDC), Ca^2+^ activated K^+^ channel (Kca), Voltage-gated potassium channels (Kv), Store-operated calcium entry (SOCE).

Since the novel coronavirus pneumonia epidemic in 2019, the mechanism of the protective effect of traditional Chinese medicine on the lungs has received much concern. Tan IIA is one of the fat-soluble substances extracted from the traditional Chinese medicine *S. miltiorrhiza Bunge*. With the deepening of research on Tan IIA around the world, more and more studies have used various *in vivo* and *in vitro* respiratory disease models to elucidate the target and potential mechanism of Tan IIA against respiratory diseases. Notably, in different *in vivo* and *in vitro* respiratory disease models, the active concentrations and action durations of Tan IIA and its derivatives vary. In in - vitro environments, Tan IIA has a wide range of active concentrations and usually a short action time. In *in vivo* murine models, the typical active concentration of Tan IIA is 10–30 mg/kg. In asthma and acute lung injury models, its action duration is relatively short (mostly 3–5 days), while for other diseases, a longer action time is required. For example, it is 1 week or longer for chronic obstructive pulmonary disease (COPD), mostly 3–4 weeks for pulmonary hypertension (PH), and 4 weeks or more for pulmonary fibrosis (PF). These differences in concentration and duration suggest that Tan IIA may be involved in different physiological and pathological processes in various respiratory diseases. This not only calls for further exploration but also provides further guidance for the clinical application of Tan IIA in combating respiratory diseases. For the above respiratory diseases except lung cancer, Tan IIA can inhibit the release of pro-inflammatory factors and oxidants, regulate ion channels, inhibit apoptosis, reduce collagen deposition, and inhibit lung EMT and FMT changes, thereby reducing lung injury and improving lung function (Figure 3). As for the most studied lung cancer, Tan IIA can not merely inhibit the growth of cancer cells, arrest the cell cycle, inhibit cancer cell migration and invasion, promote cancer cell apoptosis, play a synergistic role with anti-tumor drugs, but also reduce the resistance of drug-resistant cancer cells to drugs.

While there is great potential for studies of Tan IIA against respiratory diseases, there remain limitations that need to be addressed. First, the exact mechanism of Tan IIA against respiratory diseases needs to be further illustrated. Many studies have now reported that Tan IIA regulates macrophage reprogramming (Li and Xu, 2024) and glycolysis (Yu et al., 2024), and these mechanisms play an important role in the process of combating respiratory diseases. However, the specific molecular pathways and the interrelationships among the targets remain unclear, and it is also impossible to determine whether there are other potential actionable pathways of Tan IIA. Second, the research on Tan IIA for respiratory diseases still has limited models. The research on common clinical diseases such as pulmonary infection and bronchiectasis is relatively scarce. Future efforts should be focused on improving the research on Tan IIA in these diseases to expand its application range and gain a deeper understanding of its mechanism of action. Furthermore, Tan IIA is still in preclinical studies against respiratory diseases, and the optimal dose and duration of its treatment, as well as whether it interacts with other drugs and its safety, still need to be explored. Currently, the number of randomized controlled clinical trials and application literature on Tan IIA for respiratory diseases is relatively small, and the sample size is generally small. For example, a certain efficacy was achieved in 5 patients with PH treated with STS alone or in combination with sildenafil (Wang et al., 2013b). However, both the sample size and the scope of these studies are limited. Therefore, it is necessary to conduct multi-center, large-sample randomized controlled trials to evaluate its reliability and optimize its application strategy in the treatment of lung diseases. Finally, based on the fact that Tan IIA is a fat-soluble substance, how to improve the bioavailability of Tan IIA in the respiratory system remains to be studied. For example, new drug delivery methods such as nanomedicines (Scherliess, 2019) and aerosol administration (Woodward and Fromen, 2024) have been proposed recently for the treatment of respiratory diseases. Determining the optimal combination of Tan IIA with these administration methods to augment its bioavailability represents a persistent and challenging problem that demands continuous investigation and resolution.

Although there are still many problems to be solved urgently, it is believed that Tan IIA will be a promising drug against respiratory diseases as research gradually deepens.
